# Perfil de Derivación de Pacientes Adultos Atendidos en la Consulta de Neuropsicología del Hospital Universitario la Paz de Madrid Entre 2018 y 2023

**DOI:** 10.31083/RN36419

**Published:** 2024-12-18

**Authors:** Julia Castellanos Segado, Cristina Campoy Lacasa, Diego Carracedo Sanchidrián, Jesús Martí Esquitino

**Affiliations:** ^1^Servicio de Psiquiatría, Psicología Clínica y Salud Mental, Instituto de Investigación Hospital Universitario La Paz, Hospital Universitario la Paz (Madrid), 28046 Madrid, España; ^2^Dirección General de Salud Mental. Servicio Murciano de Salud, 30100 Murcia, España

**Keywords:** evaluación neuropsicológica, neurociencia cognitive, neuropsicología, sistema nacional de salud, trastornos neurocognitivos, cognitive neuroscience, national health system, neurocognitive disorders, neuropsychological assessment, neuropsychology

## Abstract

**Introducción:**

La neuropsicología es una rama de la psicología que estudia la relación entre el sistema nervioso, las funciones cognitivas y el comportamiento. Este trabajo pretende describir el perfil de pacientes atendidos en la consulta de neuropsicología del Hospital Universitario La Paz (HULP) entre 2018 y 2023.

**Sujetos y métodos:**

Estudio observacional retrospectivo de 394 registros clínicos de sujetos mayores de 18 años evaluados en la consulta de neuropsicología del HULP entre 2018 y 2023. Los datos se registraron, anonimizados, en una base de datos debidamente custodiada. Se extrajeron tablas de frecuencias y se realizaron pruebas χ^2^. Para el análisis de los datos se utilizó SPSS 26.

**Resultados:**

Fueron evaluadas 232 mujeres (media de edad 46,47 años) y 162 hombres (media de edad 43,31 años). El motivo de consulta más frecuente en ambos grupos fue la sospecha de demencia (112 y 76 respectivamente). Para todos los motivos de consulta existen más pacientes que estudiaron hasta los 18 años. El análisis binomial revela una relación estadísticamente significativa entre ser evaluado en la consulta de neuropsicología del HULP y haber finalizado los estudios antes de los 18 años.

**Conclusiones:**

El perfil de paciente atendido habitualmente en la consulta de neuropsicología del HULP es una mujer de mediana edad con estudios hasta los 18 años, derivada por el servicio de Salud Mental por sospecha de demencia. La información obtenida tras este análisis ayudará a planificar futuras estrategias de evaluación para los pacientes con problemas neuropsicológicos.

## Abstract (en)

**Introduction:**

Neuropsychology is an area of psychology that studies, through different methodologies, the relationship between the nervous system and behavior. This study aims to describe the profile of the patient who most frequently received a neuropsychological assessment at HULP between 2018 and 2023.

**Patients and Methods:**

This is a retrospective observational study of 394 clinical records of subjects aged 18 years or older who were attended in the neuropsychology consultation of HULP between 2018 and 2023. The data was recorded and anonymized in a database properly guarded. Frequency tables were extracted and χ^2^ tests were made. SPSS 26 was the programme used to make data analysis.

**Results:**

A total of 232 women (mean age of 46,47 years) and 162 men (mean age 43,31 years) were evaluated. The most frequent reason for consultation in both groups was suspicion of dementia (112 consultations for this reason in the female group; 76 in the male group). Regardless of the reason for consultation, there were more cases of patients who had studied up to the age of 18 years. Binomial analysis reveals a statistically significant relationship between being assessed in the neuropsychology consultation at HULP and having completed studies before the age of 18.

**Conclusions:**

The profile of the patient who is most frequently attended at the neuropsychology consultation of HULP is a middle-aged woman with education up to 18 years of age, referred by the Mental Health Service for suspected dementia. The information obtained from this analysis will help to guide future planning of assessment strategies for patients with neuropsychological problems.

## 1. Introducción

La neuropsicología es un área de la psicología que estudia la relación entre el sistema nervioso y el comportamiento, incluyendo este último las áreas psicosocial, cognitiva, conductual y emocional [1]. Utiliza métodos tanto experimentales como clínicos, que se ofrecen a la comunidad como parte del servicio de salud, siendo los más importantes la evaluación y la rehabilitación neuropsicológica [2].

La exploración neuropsicológica sirve para valorar las funciones cognitivas (atención, orientación, memoria, gnosias, funciones ejecutivas, praxias, lenguaje, cognición social y habilidades visoespaciales) y requiere de la utilización de test, pero también de la valoración cualitativa y clínica del paciente, así como de información de terceros [3]. Posteriormente, los resultados se comparan con el rendimiento normativo esperado según el grupo de referencia del paciente para cuantificar, si existe, el deterioro [4].

Hasta 1950 su función principal era el diagnóstico diferencial entre cuadros orgánicos y funcionales. En la actualidad, puede definir el efecto directo del daño cerebral y los factores psicológicos derivados de él [5], cuantificar cambios significativos en medidas repetidas [6] y aportar herramientas útiles para la detección del deterioro cognitivo cuando no existe daño neurológico [5]. En definitiva, los grupos de población para los que la evaluación puede ser útil actualmente son mucho más numerosos que los considerados años atrás [4].

Asimismo, la valoración de las funciones cognitivas no tiene un único objetivo, pudiéndose diferenciar: objetivo diagnóstico, rehabilitador, orientado a la planificación/orientación, pericial [7], restablecimiento de capacidad laboral o incremento de la calidad de vida del paciente [3], entre otros.

En España, las enfermedades que afectan al cerebro suponen un coste estimado de 84 mil millones de euros/año [8]; y son cada vez más prevalentes [9, 10]. En 2019, el número mundial de personas con demencia era 57,4 millones, lo que supone un aumento de prevalencia respecto a la última década por el aumento del envejecimiento y el crecimiento de la población [11]. De hecho, el número de personas con demencia se duplica cada cinco años, siendo la prevalencia mayor en mujeres (788 casos versus 561 casos por cada 10.000 habitantes) [12]. Además, se estima que para 2030 el coste de las demencias será abrumador para el sistema social y sanitario y, de hecho, este coste ya ha incrementado más del 35% en los últimos años [13, 14].

El Hospital Universitario La Paz (HULP) ha contado con profesionales de la neuropsicología desde los años 70, y se ha desarrollado este campo en un amplio número de patologías y servicios.

Este estudio tiene como objetivo analizar las características de los pacientes valorados en la consulta de neuropsicología clínica del HULP entre 2018 y 2023. Hasta la fecha no se cuenta con ningún análisis objetivo similar, elemental para planificar la respuesta a la creciente demanda en los hospitales.

## 2. Material y Métodos

### 2.1 Diseño

Se realiza un estudio observacional retrospectivo de registros clínicos. Para la elaboración de este artículo se ha seguido la guía de recomendaciones RECORD [15].

### 2.2 Sujetos

Se incluyeron sujetos mayores de 18 años que fueron evaluados en la consulta de neuropsicología de adultos del HULP entre 2018 (año en que comenzó el registro sistemático en una base de datos normalizada) y 2023 por psicólogos internos residentes del hospital, cuyo registro se guarda en el archivo. Las valoraciones fueron supervisadas indirectamente por un Facultativo Especialista en Psicología Clínica con formación reglada en neuropsicología. Se tomaron como criterios de exclusión el no haber finalizado la evaluación o la ausencia de datos en alguna de las variables analizadas.

### 2.3 Procedimiento

Los participantes fueron reclutados tras haber sido derivados a la consulta de neuropsicología por facultativos especialistas del hospital, y sus datos fueron recogidos por los residentes en una base de datos debidamente protegida.

#### 2.3.1 Variables e Instrumentos

Se creó una nueva base de datos con las siguientes variables: edad, sexo, motivo de derivación, servicio derivante, años de educación y año de evaluación.

Para la variable a “*motivo de derivación*”, se agruparon los datos iniciales en ocho categorías diagnósticas contempladas en el manual diagnóstico CIE-11 [16]. Así, bajo el término “*demencia*” se incluyeron los motivos de derivación de la base de datos original codificados como: “*sospecha de deterioro cognitivo/demencia, quejas de memoria y atención, alteraciones del discurso, bradipsiquia, reevaluación deterioro cognitivo, alteraciones de perfil frontal, diagnóstico diferencial Parkinson-Lewy*” y similares. Por su parte, en “*discapacidad intelectual*” se enmarcaron los motivos de consulta redactados como: “*valoración de capacidad intelectual*” y análogos. En “*epilepsia*” se contemplaron los codificados como “*evaluación pre/post de cirugía de epilepsia, epilepsia*” y equivalentes. En la categoría “*post covid*” se agruparon derivaciones inicialmente codificadas como “*post-covid, quejas post-covid, sospecha de deterioro tras covid-19*” y similares. La etiqueta “*salud mental*” se refiriere a motivos de derivación que implicaban dificultades cognitivas en contexto de algún diagnóstico de salud mental, siendo los más frecuentes: “*sintomatología afectiva, Síndrome de fatiga crónica, fibromialgia, fallos cognitivos en Trastorno por estrés postraumático (TEPT), trastorno de personalidad, síntomas disociativos*” y equivalentes. El término “*trastornos del neurodesarrollo*” recoge aquellas derivaciones descritas como “*posible Trastorno por déficit de Atención e Hiperactividad (TDAH), sospecha Trastorno del Espectro Autista (TEA), diagnóstico diferencial TEA-TDAH, dificultades de aprendizaje*” y términos comparables. La categoría “*otros*” alude a aquellos motivos de derivación no encuadrables en las demás categorías. Por último, el término “*inespecífico*” se refiere a los motivos de consulta que, por su redacción confusa, impedían comprender cuál era realmente el motivo de derivación.

La variable “*fecha de evaluación*” se transformó a “*año de evaluación*”. La variable “*edad de finalización de estudios*” se dicotomizó en “≥*18*” y “<*18*” con el fin de facilitar el análisis, haciendo referencia a si los pacientes estudiaron hasta los 18 años o continuaron estudiando desde esa edad en adelante.

#### 2.3.2 Análisis Estadísticos

Se realizaron análisis descriptivos (recuentos y proporción para variables dicotómicas, medias, desviación y rango estándar para variables continuas), así como pruebas χ^2^ para las variables cualitativas. Para estos análisis se utilizó SPSS 26 (IBM Corporation, Armonk, NY, USA).

Este estudio fue revisado y aprobado por el CEIC del HULP el 19 de octubre de 2023.

## 3. Resultados

**Fig. 1.**
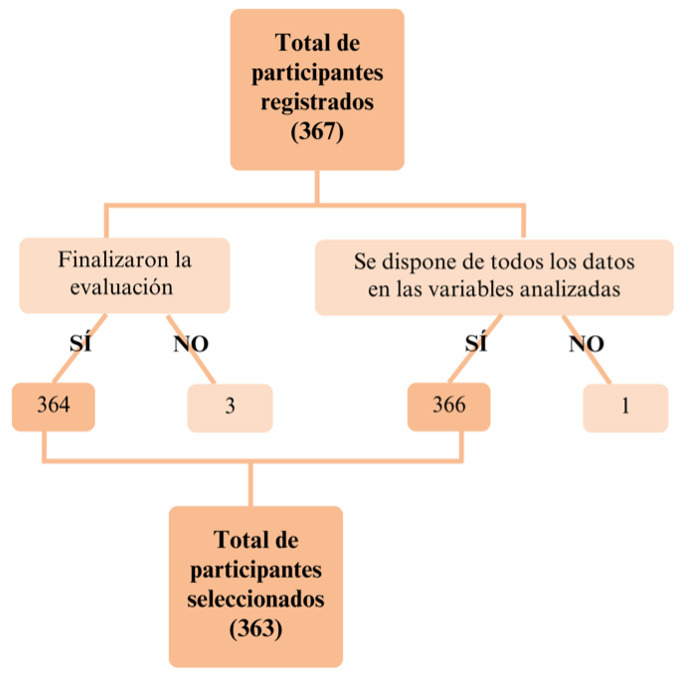
**Proceso de selección de la muestra**.

Se encontraron 398 registros de pacientes de la consulta en el periodo seleccionado, de los cuales 395 tenían datos de las variables estudiadas, tres no finalizaron la evaluación y uno no tenía datos en las variables analizadas (Fig. 1). Se realizó un análisis estadístico de los datos recogidos en la base de datos de los 394 pacientes evaluados, que fueron anonimizados previamente de manera que se asignó un código específico a cada participante que solo tendrá su correspondencia con la base de datos original en un archivo aparte, custodiado por el hospital.

**Fig. 2.**
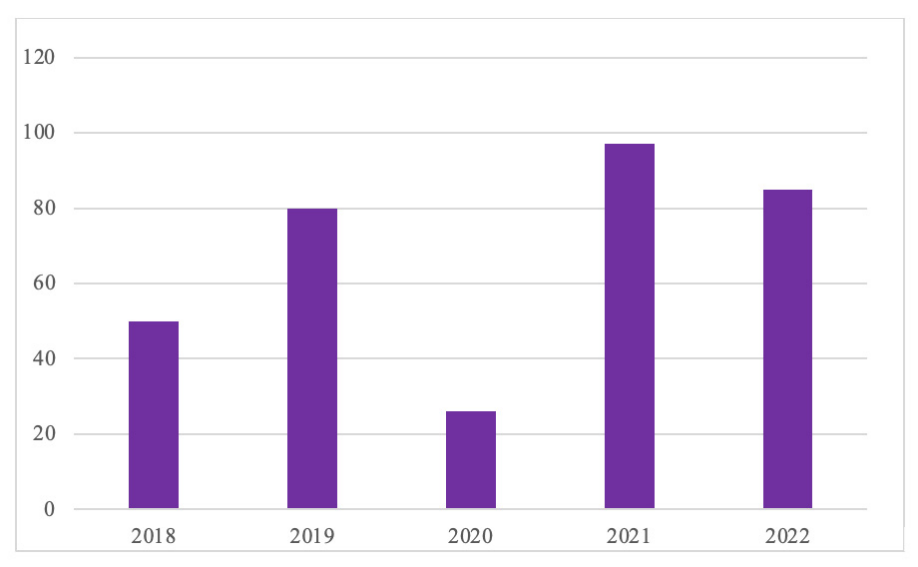
**Nº de pacientes atendidos por año**.

La Fig. 2 muestra la evolución de los 394 pacientes atendidos desde 2018 hasta 2023. Debido a la pandemia, en 2020 se priorizaron otras necesidades asistenciales, lo que llevó al cierre de la consulta durante un trimestre y a una reapertura gradual. Como resultado, disminuyó el número de pacientes atendidos.

La Tabla 1 muestra la distribución por sexo, edad y nivel educativo. El 73,35% de la muestra estudió hasta los 18 años. El análisis binomial revela una relación estadísticamente significativa entre ser evaluado en la consulta de neuropsicología del HULP y haber finalizado los estudios antes de los 18 años (*p* < 0,001). No se encuentra una relación estadísticamente significativa entre las variables sexo y nivel educativo en los análisis estadísticos bivariantes (*p* = 0,463).

**Table 1.** **Distribución por sexo, edad y nivel educativo**.

	Sexo		Edad			Edad de finalización de estudios			
						< *18*		≥ *18*	
	*N*	*%*	*M*	*DT*	*Rango*	*N*	*%*	*N*	*%*
Mujeres	232	58,88	46,47	16,28	17–81	167	42,70	65	16,60
Hombres	162	41,12	43,31	16,21	17–81	122	30,65	40	10,05
Total	394	100	45,17	16,37	17–81	289	73,35	105	26,65

M, media; DT, desviación típica.

La Tabla 2 muestra las características sociodemográficas de la muestra en función del motivo de consulta. Se aprecia en la variable sexo una distribución heterogénea, observándose un mayor número de mujeres en todos los motivos de consulta. No obstante, esta diferencia no resulta estadísticamente significativa (*p* = 0,27). Analizando su relación con el motivo de consulta mayoritario, demencia, tampoco se observan diferencias estadísticamente significativas entre hombres y mujeres (*p* = 0,79).

**Table 2.** **Datos sociodemográficos**.

Motivo de consulta	Sexo				Promedio de edad
	Hombre		Mujer		
	*n*	*%*	*n*	*%*	
Post-covid	1	0,25	11	2,79	51,33
Demencia	76	19,09	112	29,43	52,04
Otros	10	2,53	14	3,55	46,63
Salud mental	8	2,03	11	2,79	46,37
Inespecífico	7	1,77	7	1,77	38,43
Epilepsia	13	3,30	26	6,60	40,21
Discapacidad intelectual	30	7,51	32	8,12	35,50
Trastornos del neurodesarrollo	17	4,31	19	4,82	30,33

En relación con la edad, el promedio es menor en derivaciones por discapacidad intelectual y trastornos del neurodesarrollo, y mayor en pacientes evaluados por demencia y post-covid.

Por último, en cuanto al nivel educativo, existen más casos de pacientes que estudiaron hasta los 18 años en todos los motivos de derivación.

En la Tabla 3 se observa cómo los servicios que realizan más derivaciones son Salud Mental y Neurología, siendo el número de derivaciones del primero significativamente mayor.

**Table 3.** **Motivos de derivación desde 2018 hasta 2023**.

Servicio derivante	Número de derivaciones	Porcentaje
Salud Mental	281	71,32
Neurología	92	23,35
Medicina Interna	15	3,81
Rehabilitación	3	0,76
Dermatología	1	0,25
Prevención de Riesgos Laborales	1	0,25
Traumatología	1	0,25

**Fig. 3.**
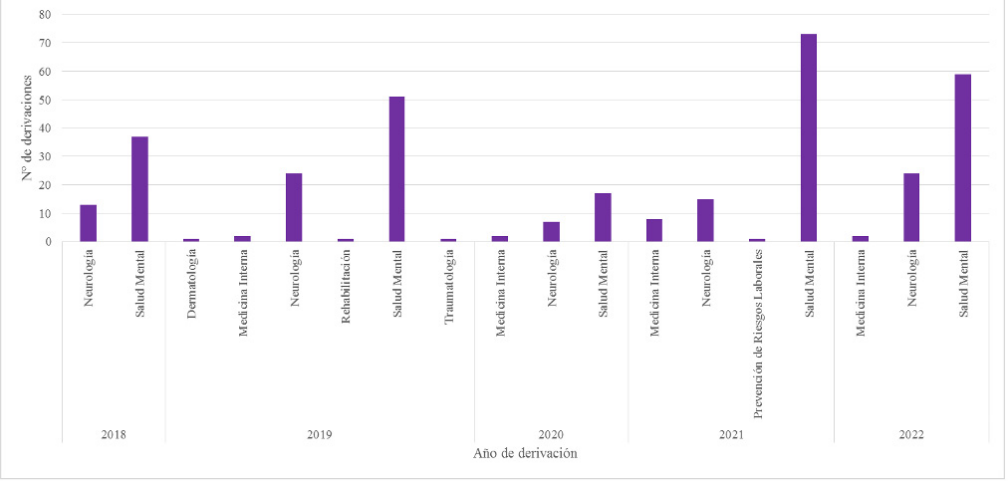
**Distribución anual de derivaciones por servicio**.

En la Fig. 3 se observa la distribución anual de derivaciones realizadas por cada servicio derivante.

## 4. Discusión

Los resultados de este análisis caracterizan la demanda de evaluación neuropsicológica en el HULP entre 2018 y 2023. En general, se puede afirmar que el paciente mayoritariamente atendido en la consulta es una mujer de mediana edad con estudios hasta los 18 años, derivada por el servicio de Salud Mental por sospecha de demencia.

Longitudinalmente se observa una demanda del servicio de evaluación neuropsicológica robusta y consolidada en los últimos años, que sugiere que esta atención es cada vez más considerada una necesidad esencial en los hospitales. Además, en el año 2021 se advierte un pico en el número de pacientes atendidos que podría ser consecuencia de la reapertura de la consulta tras la pandemia. Así, el incremento se debería a la atención a pacientes que, por el cierre de la consulta el año anterior, estaban pendientes de ser atendidos, las nuevas derivaciones recibidas en 2021 y la aparición de posibles secuelas cognitivas post-covid.

En cuanto a la distribución por sexos, como señalan investigaciones previas [17], se observó un mayor número de mujeres derivadas para todos los motivos de consulta.

En lo relativo al nivel de estudios se observó una mayoría de pacientes que estudiaron hasta los 18 años. Esto apuntaría al papel modulador y protector de la educación en la expresión de las dificultades neurocognitivas a través de múltiples e interrelacionados mecanismos [18]. Esto a su vez sugiere que el acceso a la educación y las oportunidades para el desarrollo cognitivo pueden ser esenciales, especialmente para poblaciones con discapacidad intelectual y trastornos del neurodesarrollo, quienes pueden enfrentar barreras adicionales para el acceso a un nivel educativo superior sin las oportunidades necesarias [19, 20, 21].

Para el motivo de derivación demencia no se observan diferencias significativas en el nivel educativo de los pacientes, contradiciendo esto a estudios anteriores que indican un menor riesgo de padecer un proceso neurodegenerativo en personas con niveles educativos altos [18, 22, 23]. Este resultado podría explicarse por varios factores. En primer lugar, se recogen derivaciones por sospecha de demencia, que en muchos casos no se terminarían diagnosticando tras la evaluación. Además, esta muestra incluye únicamente pacientes del área que pertenece al HULP y no de todo el territorio nacional.

Por otro lado, cabe señalar en relación con la edad que el promedio fue menor en pacientes derivados por discapacidad intelectual y trastornos del neurodesarrollo, y mayor en pacientes evaluados por demencia y post-covid, siendo estos datos coherentes con la distribución por edad habitual de dichos diagnósticos [17, 24, 25].

Al analizar los motivos de consulta se apreció que la mayoría de derivaciones fueron por sospecha de demencia, si bien no de forma estadísticamente significativa. A este respecto la literatura arroja resultados contradictorios, existiendo autores que indican un incremento preocupante de la prevalencia de las demencias [12], frente a otros que señalan un llamativo descenso últimamente [26].

En cuanto a los servicios derivantes, se hipotetiza que la gran diferencia entre las derivaciones realizadas por el servicio de Salud Mental y el resto de servicios se relaciona con varios factores. Primero, los profesionales de Salud Mental están más familiarizados con la disponibilidad y el funcionamiento de la consulta de neuropsicología, que pertenece al propio servicio. Además, para la mayoría de servicios médicos, la cognición puede no ser una prioridad o podrían desconocer que tienen la opción de derivar a la consulta si el caso lo requiriera. Por otro lado, servicios como Neurología o Geriatría cuentan con profesionales externos dedicados a la valoración neuropsicológica y además algunos de los propios facultativos tienen formación y experiencia en valoraciones mediante screening que muchas veces son suficientes. Por ello, sería recomendable ampliar la visibilidad de la consulta de neuropsicología al resto de especialistas.

Este estudio cuenta con algunas limitaciones: en primer lugar, la muestra se ha visto reducida porque existe un número indeterminado de pacientes que fueron evaluados, pero no registrados en la base de datos. Sin embargo, se asume que la pérdida sería proporcional en todos los casos, no afectando a la distribución total. En segundo lugar, el formato de registro de los datos no incluye medidas cualitativas relevantes, como el diagnóstico posterior a la evaluación, que habrían permitido extraer conclusiones de interés. Además, la edad de finalización de estudios no ha sido registrada de forma homogénea en la base de datos original.

Para superar estas limitaciones, en investigaciones futuras se sugiere registrar el diagnóstico tras la evaluación. Asimismo, se propone homogeneizar el registro cuantitativo de los datos para realizar un análisis cuantitativo e incluir datos de estudios similares en otros países. Por último, resultaría de interés realizar un seguimiento de aquellos casos con juicio clínico dudoso de forma longitudinal para obtener información adicional, especificar un diagnóstico y elaborar un plan terapéutico ajustado.

### Conclusiones

La información obtenida tras este análisis puede ayudar a guiar la planificación de estrategias de evaluación para los pacientes con problemas neuropsicológicos. Además, pone de manifiesto la importante demanda de evaluación neuropsicológica actualmente y puede contribuir a la creación de un mapa de necesidades que facilitaría la adaptación de los recursos disponibles. Se destaca la necesidad y utilidad de la consulta de neuropsicología en el Sistema Nacional de Salud, que contribuye al cumplimiento del objetivo establecido por la OMS en mayo de 2022 de “*proporcionar diagnósticos, tratamientos y atención efectivos, oportunos y con capacidad de respuesta y aplicar estrategias de promoción y prevención*” a los trastornos neurológicos. Cubriéndose así el campo de la prevención y del tratamiento, se podría reducir el coste económico que supone esta prevalente problemática [27, 28, 29]. En esta labor de detección precoz y prevención es fundamental el establecimiento de relaciones entre variables para posibilitar la localización de riesgos, para lo que este análisis sienta las bases.

## Data Availability

Los conjuntos de datos utilizados y/o analizados durante el estudio actual están disponibles a través del autor de correspondencia, previa solicitud razonable.

## Contribuciones de los Autores

JCS, CCL, DCS y JME diseñaron el estudio. JCSegado y CCL realizaron la recogida de datos, depuración de estos y revisión de la literatura. DCS revisó la recogida de datos y depuración de estos. JME realizó los análisis estadísticos. JCS, CCL y DCS redactaron el artículo. JME revisó el artículo. Todos los autores leyeron y aprobaron el manuscrito final. Todos los autores han participado lo suficiente en el trabajo y acordaron ser responsables de todos los aspectos del trabajo.

## Aprobación Ética y Consentimiento Informado

Este estudio se ha llevado a cabo cumpliendo los principios éticos establecidos en la Declaración de Helsinki y las normativas locales aplicables. El estudio fue aprobado por el comité de ética del Hospital Universitario la Paz con fecha 19 de octubre de 2023 código 2023.733; PI-5898. En este estudio retrospectivo no experimental, el comité de ética determincó que no era necesario que los participantes firmaran formularios de consentimiento informado, ya que la investigacicón involucraba datos previamente recolectados que no requercían interaccicón directa con los participantes. Esta decisicón estcá alineada con las pautas céticas estándar para estudios que utilizan datos anonimizados o preexistentes, donde obtener el consentimiento informado a menudo se considera innecesario.

## Financiación

Esta investigación no recibió financiación externa.
